# Presenteeism and musculoskeletal symptoms among nursing professionals^1^

**DOI:** 10.1590/1518-8345.2185.3006

**Published:** 2018-05-07

**Authors:** Heloisa Ehmke Cardoso dos Santos, Maria Helena Palucci Marziale, Vanda Elisa Andres Felli

**Affiliations:** 2 Msc. in Nursing. Escola de Enfermagem de Ribeirão Preto, Universidade de São Paulo, PAHO/WHO Collaborating Centre for Nursing Research Development, Brazil.; 3 PhD. Full Professor. Escola de Enfermagem de Ribeirão Preto, Universidade de São Paulo, PAHO/WHO Collaborating Centre for Nursing Research Development, Brazil.; 4 PhD. Senior Professor, Nursing School, Universidade de São Paulo, Brazil.

**Keywords:** Cumulative Trauma Disorders, Efficiency, Presenteeism, Occupational Health, Nursing, Work

## Abstract

**Objective:**

to identify the prevalence of musculoskeletal symptoms in two stages (before and after six months of the first stage) and its association with presenteeism among nursing professionals.

**Method:**

longitudinal study with quantitative data conducted in a Brazilian teaching hospital with 211 nursing professionals. The instruments used for data collection were: Cultural and Psychosocial Influences on Disability - CUPID Questionnaire, used to identify the musculoskeletal symptoms and the Stanford Presenteeism Scale, used to verify presenteeism. The instruments were validated for Brazilian Portuguese. The study was approved by the Human Research Ethics Committee. Descriptive statistics, Mann Whitney test and regression analysis were used to analyze the data.

**Results:**

158 (74.9%) professionals experienced presenteeism and 151 (71.6%) reported low back pain as musculoskeletal symptom. Professionals with low back pain had lower scores on the presenteeism scale and shoulder pain was related to loss of concentration during work.

**Conclusion:**

presenteeism lead to a reduction in work performance and was manifested in the presence of musculoskeletal symptoms. In addition, shoulder pain caused loss of concentration at work.

## Introduction

Musculoskeletal disorders are important public health problems in several countries. These disorders can cause functional limitations in adults^1^ and may interfere with work and daily life activities, arouse feelings such as impotence, uselessness, abandonment and failure, and generate costs, decrease in or lack of productivity and job losses^2^ ^-^ ^3^.

Studies have shown a prevalence of musculoskeletal symptoms higher than 70.0% among nursing professionals^4^ ^-^ ^5^. In Brazil, this prevalence is above 80.0%^6^ ^-^ ^7^. Among the musculoskeletal symptoms, pain is the most prevalent in nursing professionals, according to studies^5^ ^,^ ^7^.

Nursing professionals are exposed to occupational hazards that may compromise physical and mental health^8^, interfere with the quality of life of the worker and the quality of care provided to the patient^9^ and cause illness, absenteeism, presenteeism and generate costs for the institutions^10^.

Absenteeism is defined as the employee’s absence from work, consisting of the period the worker is absent due to some intervening motive. It is related to the frequency or duration of the work time lost when the worker does not attend and it corresponds to the absences when he was expected to be present^11^.

Presenteeism is the condition in which professionals attend the workplace and perform their activities in a non-productive way and without providing a good performance due to diseases and/or problems related to work^12^. It may be related to physical and psychological factors^13^.

This phenomenon has been a cause of concern among the working population. Among the nursing staff, it is considered a poorly diagnosed contemporary problem, which can lead to serious consequences and risks for professionals, for the institution and for healthcare users^14^.

Presenteeism affects the quality of work, since it leads to errors and omissions in tasks. It is also considered one of the risk factors for future absenteeism due to illness^10^ and it causes restriction in labor productivity, not only in relation to quantity, but also in issues related to the quality of the work produced^15^ ^-^ ^16^. It can be caused by health problems such as stress, influenza, cold, allergy, asthma and musculoskeletal pain, which often interfere with work productivity^16^. Professionals in this condition are physically at work, but their attention is scattered, which can cause accidents and eventual adverse events to the patients that are under their responsibility.

Considering that musculoskeletal problems are common among nursing professionals, that presenteeism in nursing work has already been evidenced in other studies^10^ ^,^ ^17^ ^-^ ^18^ and that preventive measures are necessary to minimize these problems, we are motivated to find answers to the following question: do musculoskeletal symptoms in nursing professionals cause presenteeism? Therefore, this study was developed with the objective of identifying the prevalence of musculoskeletal symptoms in two stages (before and after six months of the first stage) and its association with presenteeism among nursing professionals.

## Method

Longitudinal study with quantitative data conducted with nursing assistants, nursing technicians and nurses in a teaching hospital in Ribeirão Preto/SP - Brazil. The study sample was based on the presence of musculoskeletal pain, considering the region of the body that presented the highest value. To calculate the sample, a pilot study was carried out with 30 nursing professionals, ten from each category (nursing assistant, nursing technician and nurse) and information on the prevalence of musculoskeletal pain and participant loss (professionals who refused to participate on the second stage of the study) were obtained. The method used was stratified sampling and the professional categories were used as stratification variables.

The participants included in the study were nursing professionals who had been working at the hospital for at least one year and who were between 20 and 59 years old, excluding those who were on vacation and who turned 60 in April 2015. The inclusion criterion is based on other studies carried out by the main author of the *Cultural and Psychosocial Influences on Disability - CUPID Questionnaire,* in which there is a greater amount of professionals in labor activity.

Data collection was performed in two stages. The first stage occurred between May and June 2015 using the CUPID Questionnaire^19^ ^-^ ^20^, validated for the Brazilian Portuguese^19^. with the objective of identifying demographic, occupational characteristics and musculoskeletal symptoms in the last twelve months (baseline questionnaire) and last month (follow-up questionnaire) among nursing professionals. The interval between the first and second stage was of six months, due to the reduced time for implementation of the study. The *Stanford Presenteeism Scale - SPS* 6^10^ validated for Brazilian Portuguese was used to evaluate general presenteeism at work through the factors “completing work” and “avoiding distraction”. The issues associated with completing work refer to the amount of work that is accomplished when the worker is under the influence of the causes of presenteeism, manifested through physical symptoms. The issues regarding avoiding distraction correspond to the capacity of concentration that the professionals present when symptoms of presenteeism are manifested. This instrument indicates how health circumstances and problems affect the productivity of each worker, and considers that each individual has different ways of reacting to and overcoming the symptoms caused by illness, resulting in different degrees of physical and/or mental impairment for the individual performance at work^10^ ^,^ ^21^. *SPS - 6* was applied only to professionals who presented pain in one or more regions of the body, since this scale evaluated how much the presence of this pain interfered in the work.

The second stage was conducted in November and December 2015. In this stage, the follow-up questionnaire part of the CUPID Questionnaire^19^ was applied in the sample of nursing professionals who participated in the first stage and accepted to participate in the second, with the objective of collecting information similar to the baseline questionnaire within a period of approximately six months after completion of the first one. At this stage, information regarding demographic, occupational data and presence of pain, in each of the six anatomical regions (lower back, neck, shoulder, elbow, wrist/hand and knee) during the last month were assessed ^(^ ^19^.

The CUPID Questionnaire data was analyzed through percentage values and the data from SPS 6 was analyzed based on the recommendations of the main author of the instrument: the total score of the SPS-6 is composed of the sum of the score ​​of the scale items, which can range from 6 to 30, so a low score (6 to 18) indicate reduced performance and high scores (close to or equal to 30) indicate a greater ability of the worker to concentrate and perform his work despite the health problem^10^ ^,^ ^21^.

The data collected were stored in a database in the program Microsoft Office Excel version 2010, and later entered in the statistical analysis program Statistical Package for Social Science (SPSS) version 22 and in the program R version 3.1.2. Descriptive statistics, the Mann Whitney test and multiple linear regression truncated in the interval of six to 30 according to the score ​​of SPS - 6^22^ were used for data analysis and are presented in tables. In all analyzes the level of significance (alpha) adopted was 5% (0.05).

This study followed all the ethical recommendations for research with human beings and good practices in research^23^. There was no conflict of interest and the research project was approved under CAAE protocol: 37430614.0.0000.5393 from the Research Ethics Committee of Ribeirão Preto College of Nursing, University of São Paulo, Brazil. The questionnaires were given to the nursing professionals who consented to participate in the study and signed the informed consent form. All questionnaires were self-completed out of working hours and lasting approximately 30 minutes, as recommended by the main author.

## Results

A total of 348 nursing professionals were invited to compose the sample. From these, 211 professionals (60.6%) accepted to participate in the first stage of the study, of which 134 (63.5%) signed, in written, their intention and consent to participate in the second stage of the study. However, when the second stage data collection was performed, 90 nursing professionals (67.2% of the 134 sample) actually answered the data collection instrument.

Regarding the losses in the first stage, 62 (17.8%) refused to participate, 31 (8.9%) did not work in the institution at the time of data collection, 23 (6.6%) were on leave and 21 (6.0%) were on vacation.

Of the 211 (60.6%) professionals who participated in the first stage, 175 (82.9%) were women, 36 (17.1%) were men and the mean age was 42.3 years. Regarding the occupational variables, 106 (50.2%) were nursing assistants, 53 (25.1%) were nursing technicians and 52 (24.7%) were nurses. Regarding the demographic data of the 90 professionals who participated in the second stage, 74 (82.2%) were female, 16 (17.8%) were male, 45 (50.0%) were nursing assistants, 21 (23.3%) were nursing technicians, 24 (26.7) were nurses and the mean age was 42.51 years.

Table 1 presents the prevalence of pain in the anatomical regions (lower back, neck, shoulder, elbow, wrist and/or hand and knee) in the last twelve months among nursing professionals in the first stage of the study.

Considering the data in Table 1, low back pain was the most common symptom among participants in the first stage of the study, manifested in 151 (71.6%) nursing professionals in the last twelve months. Neck pain was the second most mentioned pain by nursing professionals.

Table 2 shows the frequency of pain in the anatomical regions in the last month among nursing professionals in the first stage of the study.

According to table 2, 118 (78.1%) participants manifested low back pain.

Table 3 present data on the prevalence of pain among nursing professionals who participated in the second stage.

According to Table 3, low back pain (42-46.7%) and neck pain (34-37.8%) were the most reported by the 90 professionals who participated in the second stage of the study.

Table 4 presents the score ​​values of the presenteeism and the presence of pain in the last month referring to the first stage of the study.

Among the 211 nursing professionals in the sample, 163 (77.3%) presented pain in one or more anatomical regions in the last month and 48 (22.7%) did not report this symptom in this period. Of the 163 participants with pain, five (2.4%) had pain, but did not answer the presenteeism scale, so 158 (74.9%) professionals answered the scale correctly.

According to Table 4, the results indicate that among the 158 nursing professionals, 115 (72.8%) reported pain in the lower back, 91 (57.6%) neck pain, 74 (46.8%) pain in the shoulder, 25 (15.8%) pain in the elbow, 52 (32.9%) pain in the wrist and/or hand and 66 (41.8%) pain in the knee.

**Table 1:** Distribution of nursing professionals working in the teaching hospital according to the presence of musculoskeletal pain in the last twelve months (first stage). Ribeirão Preto-SP, Brazil, 2015

Variables	Twelve months					
	Lower back n(%)	Neck n(%)	Shoulder n(%)	Elbow n(%)	Wrist and/or hand n(%)	Knee n(%)
Yes	151(71.6)	112(53.1)	92(43.6)	29(13.7)	67(31.8)	79(37.4)
No	60(28.4)	99(46.9)	119(56.4)	182(86.3)	144(68.2)	132(62.6)
Total	211(100.0)	211(100.0)	211(100.0)	211(100.0)	211(100.0)	211(100.0)

**Table 2:** Distribution of nursing professionals working in the teaching hospital according to the presence of musculoskeletal pain in the last month (first stage). Ribeirão Preto-SP, Brazil, 2015

Variables	Last month					
	Lower back n(%)	Neck n(%)	Shoulder n(%)	Elbow n(%)	Wrist and/or hand n(%)	Knee n(%)
Yes	118(78.1)	92(82.1)	76(82.6)	26(89.7)	52(77.6)	67(84.8)
No	33(21.9)	20(17.9)	16(17.4)	03(10.3)	15(22.4)	12(15.2)
Total	151(100.0)	112(100.0)	92(100.0)	29(100.0)	67(100.0)	79(100.0)

**Table 3:** Distribution of nursing professionals working in the teaching hospital according to the presence of musculoskeletal pain in the last month/after six months (second stage). Ribeirão Preto-SP, Brazil, 2015

Variables	Last month/after six months					
	Lower back n (%)	Neck n (%)	Shoulder n (%)	Elbow n (%)	Wrist and/or hand n (%)	Knee n (%)
Yes	42(46.7)	34(37.8)	30(33.3)	13(14.4)	28(31.1)	27(30.0)
No	48(53.3)	56(62.2)	60(66.7)	77(85.6)	62(68.9)	63(70.0)
Total	90(100.0)	90(100.0)	90(100.0)	90(100.0)	90(100.0)	90(100.0)

**Table 4:** Distribution of the values ​​obtained according to the scores of presenteeism and presence of pain in the last month (baseline questionnaire) among nursing professionals working in the teaching hospital. Ribeirão Preto-SP, Brazil, 2015

Pain	Category	n (%)	Mean	Median	Minimum/Maximum	Standard Deviation (SD)	p value*
Total presenteeism							
Lower back	Yes	115(72,8)	22,26	22.0	13/30	4.16	0.7783
	No	19(12,0)	21,79	21.0	7/30	6.14	
Neck	Yes	91(57,6)	22,35	22.0	13/30	4.43	0.7933
	No	18(11,4)	21,67	21.0	7/30	5.51	
Shoulder	Yes	74(46,8)	22,16	22.0	14/30	4.10	0.4060
	No	08(5,1)	23,38	26.0	14/29	5.78	
Elbow	Yes	25(15,8)	20,80	21.0	14/29	4.67	0.8345
	No	02(1,3)	19,50	19.5	19/20	0.71	
Wrist/Hand	Yes	52(32,9)	22,02	21.5	7/30	4.62	0.8345
	No	11(7,0)	22,55	23.0	16/30	4.70	
Knee	Yes	66(41,8)	22,26	21.0	13/30	4.48	0.7264
	No	08(5,1)	20,88	20.5	7/30	7.02	
Avoiding distraction							
Lower back	Yes	115(72.8)	9.27	9.0	3/15	3.20	0.5114
	No	19(12.0)	10.05	8.0	4/15	4.40	
Neck	Yes	91(57.6)	9.35	9.0	3/15	3.39	0.7337
	No	18(11.4)	9.11	8.5	4/15	3.79	
Shoulder	Yes	74(46.8)	9.10	9.0	3/15	3.21	0.0215
	No	08(5.1)	12.0	12.5	6/15	3.02	
Elbow	Yes	25 (15.8)	8.48	7.0	3/15	3.71	0.5457
	No	02(1.3)	9.50	9.5	8/11	2.12	
Wrist/Hand	Yes	52(32.9)	9.31	9.0	3/15	3.37	0.8343
	No	11(7.0)	9.46	10.0	4/15	3.17	
Knee	Yes	66(41.8)	9.30	9.0	4/15	3.41	0.8267
	No	08(5.1)	9.50	8.5	4/15	3.96	
Completing work							
Lower back	Yes	115(72.8)	12.99	14.0	3/15	2.41	0.3027
	No	19(12.0)	11.74	13.0	3/15	3.98	
Neck	Yes	91(57.6)	13.00	14.0	6/15	2.22	0.9499
	No	18(11.4)	12.56	14.0	3/15	3.68	
Shoulder	Yes	74(46.8)	13.07	14.0	3/15	2.20	0.4325
	No	08(5.1)	11.38	12.5	4/15	4.24	
Elbow	Yes	25(15.8)	12.32	13.0	3/15	2.98	0.0789
	No	02(1.3)	10.00	10.0	9/11	1.41	
Wrist/Hand	Yes	52(32.9)	12.71	13.0	3/15	2.70	0.8241
	No	11(7.0)	13.09	13.0	9/15	1.97	
Knee	Yes	66(41.8)	12.95	14.0	3/15	2.48	0.1497
	No	08(5.1)	11.38	11.5	3/15	3.78	

*Mann-Whitney test

The mean values of the total presenteeism score related to pain ranged from 20.80 to 22.35 points; the highest score referred to neck pain and the lowest score to elbow pain. The median ranged from 21 to 22, the highest score referred to low back, neck and shoulder pain, and the lowest score to knee and elbow pain. The SD score were between 4.10 and 4.67, and the highest value represented the elbow and the lowest the shoulder.

Regarding the score ​​on avoiding distraction, the mean score varied from 8.48 to 9.35, respectively referring to pain in the elbow and in the neck. The median scores ​​ranged from 7 to 9, the lowest score referring to pain in the elbow and the higher to pain in the lower back, neck, shoulder, wrist and/or hand and knee. Regarding the SD scores, the minimum was 3.20 referring to low back pain, while the maximum was 3.71, referring to elbow pain.

The scores of the factor completing work ranged from 12.32 to 13.07, respectively representing elbow and shoulder pain. The median ranged from 13 to 14; the score 13 was related to pain in the elbow and wrist, and the score 14 represented lower back, neck, shoulder and knee pain. SD scores ranged from 2.20 to 2.98 and respectively represented shoulder and elbow pain.

The data (pain and presenteeism scores) was compared through the non-parametric Mann-Whitney test. There was difference in the mean of the factor avoiding distraction among the nursing professionals who presented shoulder pain; the mean was 9.10, median 9, minimum 3, maximum 15 and SD of 3.21.

Data from the regression analysis of total presenteeism score, pain in the last month, professional category and age are presented in Table 5.

**Table 5:** Regression analysis of total presenteeism score, pain in the last month (baseline questionnaire), professional category and age among nursing professionals working in the teaching hospital. Ribeirão Preto-SP, Brazil, 2015.

Presenteeism/Sociodemographics				
Mean parameters	Estimate	Standard error	t	p value
Intercept	22.8158	3.1301	7.2890	0.0000
Females	-1.0276	1.4710	-0.6990	0.4860
Nursing technician	-0.3720	1.3101	-0.2840	0.7770
Nurse	0.0950	1.4235	0.0670	0.9470
Age	0.0422	0.0626	0.6740	0.5020
Standard deviation	1.6795/5.3629	0.0940	17.8600	0.0000
Presenteeism/Low back pain				
Intercept	20.1017	3.5790	5.6170	0.0000
Females	-1.0204	1.5681	-0.6510	0.5160
Nursing technician	0.0636	1.3498	0.0470	0.9630
Nurse	0.1596	1.5133	0.1050	0.9160
Age	0.0698	0.0659	1.0600	0.2910
Low back pain	1.1236	1.5824	0.7100	0.4790
Standard deviation	1.6623/5.2713	0.0980	16.9500	0.0000
Presenteeism/Neck pain				
Intercept	21.4729	4.1088	5.2260	0.0000
Females	-2.8880	2.1151	-1.3650	0.1750
Nursing technician	-0.9283	1.5826	-0.5870	0.5590
Nurse	0.5416	1.7493	0.3100	0.7570
Age	0.1115	0.0808	1.3790	0.1710
Neck pain	-0.1586	1.7888	-0.0890 14.9100	0.9300 0.0000
Standard deviation	1.6984/5.4652	0.1139		
Presenteeism/Shoulder pain				
Intercept	22,2096	4,3185	5,1430	0,0000
Females	-1,5424	1,7077	-0,9030	0,3690
Nursing technician	0,8176	1,5011	0,5450	0,5880
Nurse	1,6143	2,0714	0,7790	0,4380
Age	0,0704	0,0787	0,8950	0,3740
Shoulder pain	-1,5830	2,2201	-0,7130	0,4780
Standard deviation	1,5738/4,8249	0,1166	13,5000	0,0000
Presenteeism/Elbow pain				
Intercept	20.8536	6.3959	3.2600	0.0039
Females	2.1827	2.6855	0.8130	0.4259
Nursing technician	-0.7063	2.1934	-0.3220	0.7508
Nurse	14.2367	7.6046	1.8720	0.0759
Age	-0.0753	0.1222	-0.6160	0.5450
Elbow pain	1.0330	3.1840	0.3240	0.7490
Standard deviation	1.4113/4.1013	0.1617	8.7300	0.0000
Presenteeism/Wrist and/or hand pain				
Intercept	22.4353	5.4332	4.1290	0.0001
Females	-1.6264	2.5081	-0.6480	0.5194
Nursing technician	2.8956	2.3927	1.2100	0.2314
Nurse	1.0873	2.3010	0.4730	0.6384
Age	0.0205	0.0991	0.2060	0.8372
Wrist and/or hand pain	0.6074	2.4274	0.2500	0.8034
Standard deviation	1.6880/5.4087	0.1470	11.4800	0.0000
Presenteeism/Knee pain				
Intercept	21.9099	5.0205	4.3640	0.0000
Females	-3.1231	2.3598	-1.3230	0.1900
Nursing technician	-1.7219	2.0192	-0.8530	0.3970
Nurse	0.1664	2.4716	0.0670	0.9470
Age	0.0828	0.0968	0.8560	0.3950
Knee pain	1.0125	2.5916	0.3910	0.6970
Standard deviation	1.7386/5.6894	0.1419	12.2500	0.0000

The regression analysis shown in Table 5 observed 157 participants, since one did not fill age in the questionnaire. The expected mean score of presenteeism among male professionals, nursing assistants and at zero year of age was 22.8158 points. Considering the same professional category and age, women had a score 1.0276 points lower than men. Nursing technicians had a score 0.3720 points lower than nursing assistants and nurses had a score 0.0950 points higher than nursing technicians. For the variable age, for each year of age, an average increase of 0.0422 is expected.

Presenteeism and low back pain were observed in 133 participants and the expected mean score among men, nursing assistants at zero year old was 20.1017 points. Women scored 1.0204 points less that the men, nurse technicians scored 0.0636 more than assistants, nurses scored 0.1596 points more than technicians and nursing assistants and for each year of age there was a 0.0698 increase in the presenteeism score. For participants who reported low back pain a 1.1236 increase in the mean is expected.

Presenteeism and neck pain were observed in 108 nursing professionals. For these professionals, the expected mean value for male participants, nursing assistants and at zero year of age was 21.4729 points. Among the other variables, it was identified that women presented a score 2,8800 points lower, technicians 0.9283 points lower, nurses 0.5416 points higher and for each year of age the professionals presented a 0.1115 points increase in the presenteeism score. For professionals who reported neck pain a 0.1586 decrease in the presenteeism occurred.

In the regression analysis of presenteeism and shoulder pain, 81 participants were observed. For men, nursing assistants and at zero year of age, the mean value was 22.2096. Female nursing professionals scored 1.5424 less, technicians and nurses scored 0.8176 and 1.6143 more respectively, and for each year of age, there was an increase of 0.0704. Among nursing professionals with shoulder pain, there was a 1.5830 decrease in the score.

Regarding presenteeism and elbow pain, 27 people were observed and an average score of 20.8536 was found for men, nursing assistants and at zero year of age. Women obtained 2.1827 more, nursing technicians scored 0.7063 less and nurses 14.2367 more, and for each year of age the professionals scored 0.0753 points less. For professionals who reported pain in the elbow, a 1.0330 increase in the mean of presenteeism was found.

Regarding the variables presenteeism and pain in the wrist and/or hand, 62 professionals were observed. The mean expected value of presenteeism for male professionals, nursing assistants and at zero year of age was 22.4353 points. For women, a decrease of 1.6264 points was observed. Nursing technicians and nurses presented an increase of 2.8956 and 1.0873 points respectively. For each year of age the professionals had an increase of 0.0205 points. Nursing professionals who reported pain in the wrist and/or hand in the last month obtained 0.6074 more in the mean of presenteeism.

Regarding the 74 nursing professionals observed on presenteeism and knee pain, the mean score for male professionals, nursing assistants and at zero year old was 21.9099. Considering the same professional category and age, women presented 3.1231 less than men. Nursing technicians scored 1.7219 less and nurses 0.164 higher on mean score. For each year of age, there was an increase of 0.0828. For nursing professionals who reported knee pain, a score 1.0125 higher was verified. For all variables analyzed, no significant differences were observed in relation to pain.

## Discussion

The demographic characteristics of the sample studied reflect the profile of nursing professionals in Brazil, a profession with a 84.6% prevalence of women^24^ ^-^ ^25^. The mean age of the participants was 42.3 years old in the first stage and 42.51 years old in the second, which is in accordance with other studies conducted with nursing professionals who reported musculoskeletal symptoms^25^.

The professional category with the largest number of participants in the first part of the study was nursing assistants, followed by nursing technicians and nurses. In the second stage, the priority trend was nursing assistants, nurses and nursing technicians. In addition, the presence of musculoskeletal symptoms was higher among the nursing assistants. Studies show a higher prevalence of symptoms among nursing assistants^26^, since these professionals perform several procedures, such as lifting weight, moving and cleaning patients, changing and organizing beds and others tasks that can lead to musculoskeletal symptoms^1^ ^,^ ^5^.

Among the most prevalent musculoskeletal symptoms, low back pain was the most frequently reported by nursing professionals, followed by neck pain. These results are confirmed in other studies^2^ ^,^ ^5^.

Of the nursing professionals who experienced presenteeism, the majority were female professionals. This was also observed in a study conducted in Slovenia^27^. Regarding the professional category, the presenteeism score was lower among nursing technicians.

Studies conducted to identify presenteeism among nursing professionals found a high prevalence of female professionals^17^ ^-^ ^18^ ^,^ ^20^ ^,^ ^27^, corroborating the results of this study. Regarding the professional category, a study conducted in Brazil to identify presenteeism among nursing professionals found that the majority were nursing assistants and technicians^10^; thus, the results of this study are similar to the data in the literature.

Regarding the total presenteeism score observed through the SPS-6, musculoskeletal symptoms related to presenteeism affected mostly the female professionals and the nursing technicians. According to this scale scores, the musculoskeletal problems lead to presenteeism among nursing professionals and influenced the performance of the work activities in relation to avoiding distraction and completing work, all related to the reduction of performance in the work activities. There was a statistically significant difference regarding shoulder pain and avoiding distraction. The results of the sub-dimensions of the scale showed that the men and the nursing technicians presented lower concentration at work due to musculoskeletal symptoms. In addition, these symptoms influenced the amount of work done, reducing performance.

A study conducted in a hospital in Portugal evaluated the impact on costs caused by loss of productivity and presenteeism among nursing professionals with musculoskeletal symptoms. The study found that these professionals had higher mean scores in the sub-dimension avoiding distraction compared to completing work. In addition, the nursing assistants had higher levels of presenteeism in both sub-dimensions^18^.

The present study also measured the association between presenteeism scores and presence of pain in the anatomical regions in the participating nursing professionals and found that low back pain was the most frequently reported by these professionals. Data from the literature corroborate the results of this research, indicating that low back pain is associated with presenteeism among nursing professionals^27^ ^-^ ^29^. In addition, a high prevalence of low back pain and presenteeism was identified in other studies ^18^ ^,^ ^27^ ^,^ ^29^.

The present study found elbow pain as the lowest score on the scale of presenteeism in relation to the total score and the sub-dimensions avoiding distraction and completing work. A difference in the factor avoiding distraction was found for nursing professionals who presented shoulder pain, with a mean score of 9.10. Thus, it can be confirmed that nursing professionals with shoulder pain had a lower level of concentration at work. The pain interfered negatively in the work activities of the nursing professionals, reducing their performance at work.

Through the regression analysis, it was possible to identify that nursing professionals with low back pain had a lower score on the presenteeism scale (20.1017), which indicates that the worker had low capacity to concentrate and perform the work when in presence of this pain. Similar data were found in an international study conducted in Slovenia with nursing professionals, in which the score of presenteeism related to low back pain was around 20^27^.

Female participants presented negative scores in all analysis, except for elbow pain. Besides that, knee pain scored the highest negative score among women. Therefore, it was verified that presenteeism scores were lower for women than for men.

The nurses presented positive scores related to sociodemographic variables and pain. However, nursing technicians had negative scores on the presenteeism scale for pain in the neck, elbow, knee and sociodemographic characteristics. Therefore, nursing technicians were the professionals who obtained the most negative scores on presenteism.

Age was one of the factors that influenced the presenteeism scores. The score of this variable was negative only for elbow pain and caused positive and negative association. Negative scores occurred for neck and shoulder pain, but the pain influenced the final presenteeism scores among nursing professionals.

The present study identified that musculoskeletal pain can cause presenteeism. The limitations of the study were the worker-specific composition of the sample was, limited to one hospital and the interval from one stage to another, which was of six months, a time different from other studies carried out with the CUPID questionnaire in which the interval was twelve months. In addition, the use of self-completed instruments may lead to bias and possible interference of uncontrolled factors.

## Conclusion

Among the musculoskeletal symptoms prevalent among nursing professionals, low back pain was the most frequently reported. Presenteeism occurred to a high number of nursing professionals, causing a reduction in work performance and manifesting itself in the presence of musculoskeletal symptoms. In addition, shoulder pain is related to loss of concentration during work. Future studies on this subject and on its repercussion for nursing professionals are suggested to broaden scientific knowledge and promote planning of preventive actions.
